# Abiraterone acetate for chemotherapy-naive metastatic castration-resistant prostate cancer: a single-centre prospective study of efficacy, safety, and prognostic factors

**DOI:** 10.1186/s12894-018-0416-6

**Published:** 2018-12-03

**Authors:** Liancheng Fan, Baijun Dong, Chenfei Chi, Yanqing Wang, Yiming Gong, Jianjun Sha, Jiahua Pan, Xun Shangguan, Yiran Huang, Lixin Zhou, Wei Xue

**Affiliations:** Department of Urology, School of Medicine, Renji Hospital, Shanghai Jiao Tong University, Shanghai, 200127 China

**Keywords:** Abiraterone, Metastatic castration-resistant prostate cancer, Chemotherapy-naive, Response to previous therapy

## Abstract

**Background:**

To evaluate the efficacy and safety of abiraterone acetate (AA) plus prednisone compared with prednisone alone in Asian patients with chemotherapy-naive metastatic castration-resistant prostate cancer (mCRPC), and to identify predictive factors.

**Methods:**

We reviewed the medical records of 60 patients with chemotherapy-naive mCRPC at Renji Hospital who were treated with AA plus prednisone (*n* = 43) or prednisone alone (*n* = 17). All patients were assessed for prostate-specific antigen (PSA) response, PSA progression-free survival (PSA PFS), radiographic progression-free survival (rPFS), and overall survival (OS). The ability of several parameters to predict PSA PFS, rPFS, and OS was studied.

**Results:**

The median follow-up time was 14.0 months (range 7.0–18.5 months), at which time 19 death events had been reported: 11 in the AA + prednisone group and 8 in the prednisone group. The AA + prednisone group had significantly longer median PSA PFS (10.3 vs 3.0 months, *P* < 0.001), rPFS (13.9 vs 3.9 months, P < 0.001), and OS (23.3 vs 17.5 months, *P* = 0.016) than the prednisone-alone group. The most frequently reported grade 3 or 4 adverse event in both the AA + prednisone and prednisone-alone groups was elevated alanine aminotransferase level in 5 of 43 patients (11.6%) and 2 of 17 patients (11.8%), respectively. No adverse events led to discontinuation of therapy. In multivariate analysis, time from androgen deprivation therapy (ADT) to castration resistance of ≤18 months was a determinant of shorter OS (*P* = 0.007).

**Conclusions:**

These results support the favourable safety and efficacy profile of AA for the treatment of Asian patients with chemotherapy-naive mCRPC. Longer duration of ADT response was significantly associated with longer survival.

**Electronic supplementary material:**

The online version of this article (10.1186/s12894-018-0416-6) contains supplementary material, which is available to authorized users.

## Background

Prostate cancer (PC), particularly metastatic castration-resistant prostate cancer (mCRPC), accounts for a large proportion of the global cancer burden [1]. Although most men with PC initially respond to androgen-deprivation therapy (ADT), the response is not sustained and almost all patients eventually progress to a lethal castration-resistant phenotype within 18–24 months [2].

Chemotherapy has been the standard treatment for mCRPC since 2004 [3–5]. More recently, the treatment paradigm had been dramatically altered by the advent of several AR pathway-targeted agents, new-generation chemotherapies, and immunotherapies [6]. Abiraterone acetate (AA) is a potent and selective inhibitor of CYP17, a key enzyme in androgen biosynthesis [7]. AA blocks the transformation of cholesterol to androgens in the testicles, adrenal glands, and tumor, which reduces serum testosterone to undetectable levels [7–9]. The COU-AA-301 and COU-AA-302 trials demonstrated a significantly increased median overall survival (OS) of patients with ECOG-PS (Eastern Cooperative Oncology Group performance status) scores of 0 or 1 when treated both after and before chemotherapy with AA compared with placebo [10–13].

Currently, AA is not commonly used in clinical practice in Asian countries, and little is known about the clinical outcomes of Asian populations with chemotherapy-naive mCRPC treated with AA in real-world clinical practice. Moreover, AA is usually used to treat patients whose ECOG-PS is 2 in clinical practice, unlike the setting of previous clinical trials that included only patients with ECOG-PS of ≤1 [10–13] [14]. In addition, the response rates to AA are currently unpredictable. Thus, the ability to accurately predict response to AA would facilitate clinical decision-making, especially if alternative treatments such as chemotherapy are being considered.

In the present study, we evaluated the efficacy and safety of AA in the treatment of chemotherapy-naive mCRPC patients (ECOG-PS ≤2) at our centre in China and analysed factors predicting the outcome.

## Methods

### Patient population

Sixty chemotherapy-naive mCRPC patients have written consent and were enrolled in our study between September 2012 and March 2016. We have calculated the sample size of this study based on the previous similar studies [15, 16]. Approval for this study was obtained from the Committee for Ethics of Renji Hospital. Of the 60 patients, 43 have received AA and prednisone and 17 have received prednisone alone. These patients were consecutively enrolled in this study.

Briefly, eligibility criteria were age ≥ 18 years with histologically or cytologically confirmed adenocarcinoma of the prostate; prostate-specific antigen (PSA) progression in accordance with Prostate Cancer Clinical Trials Working Group 2 (PCWG2) criteria or radiographic progression in soft tissue or bone with or without PSA progression; ongoing ADT with a serum testosterone level of < 50 ng/dL (1.7 nmol/L); previous ADT followed by PSA progression after discontinuing the anti-androgen therapy. Patients who had received therapy with ketoconazole for more than 7 days were excluded.

Patients in the AA + prednisone group received AA 1 g orally twice daily (administered as four 250 mg tablets) plus prednisone 5 mg orally twice daily. Patients in the prednisone-alone group received prednisone 5 mg orally twice daily.

### Procedures

Radiographic examination with computed tomography and bone scanning were performed every 8 weeks for the first 24 weeks and every 12 weeks thereafter. Clinical safety assessments included laboratory monitoring of blood chemistry, haematological values, kidney function, serum lipids, and PSA at baseline and at monthly visits thereafter.

### Efficacy outcomes

The definitions of PSA and radiographic progressive disease were in accordance with the PCWG-2 criteria [17]. The co-primary end points were PSA response rate (proportion of patients achieving ≥50% PSA decline according to Prostate Specific Antigen Working Group criteria), PSA progression-free survival (PSA PFS), radiographic progression-free survival (rPFS), and OS. OS was defined as the time from the first dose to the last follow-up (March 2016) or death. PSA PFS and rPFS were defined as the time from first dose to PSA progression or radiographic progression, respectively, as previously described [17], or the time to last follow-up or death.

### Safety analysis

Clinical assessments were conducted at monthly visits and included medical history, vital sign measurements, physical examination, review of concomitant therapy and procedures, and review of adverse events (AEs).

### Statistical analysis

The cut-off date for analysis was 17 March 2016. At that time, there had been 11 deaths in the AA + prednisone group (25.58%) and 8 deaths in the prednisone group (47.06%). The median follow-up time was estimated by the Kaplan–Meier method, with death as a censoring event. Continuous variables, except time from ADT to castration resistance, are reported as the medians with interquartile ranges (IQR) and were analysed by the Wilcoxon signed rank test. The time from ADT to castration resistance was dichotomised by calculating the area under the receiver operating characteristic (ROC) curve to identify the optimal cut-off time. Categorical variables were analysed by chi-square tests. Survival distributions, including PSA PFS, rPFS, and OS, were estimated by the Kaplan–Meier method, and treatment differences were compared using the log-rank test. X^2^-test was used to compare group differences in PSA response rates. An exploratory multivariate analysis for OS, PSA PFS, and rPFS was performed using the Cox proportional hazards model, adjusting for known baseline prognostic factors. All tests were two-sided. Differences were considered to be statistically significant at *P* < 0.05. SPSS version 19.0 software was used for all analyses.

## Results

### Efficacy outcomes

Sixty patients diagnosed with chemotherapy-naive mCRPC at Renji Hospital between September 2012 and March 2016 were enrolled (Raw data was shown in Additional file 1). At the time of follow-up, treatment was ongoing for 20 patients(46.5%) in the AA + prednisone group and no patients (0%) in the prednisone-alone group. The main reason for discontinuation was disease progression for both groups, and no patients stopped treatment because of side effects.

At the median follow-up time of 14.0 months (IQR: 7.0–18.5), 19 deaths had been observed: 11 of 43 patients (25.6%) in the AA + prednisone group and 8 of 17 patients (47.1%) in the prednisone-alone group. PSA progression and radiographic progression were observed in the same number of patients in the AA + prednisone group (both 22 of 43 patients [51.2%]) and in the prednisone-alone group(both 17 of 17 patients [100%]).

**Fig. 1:**
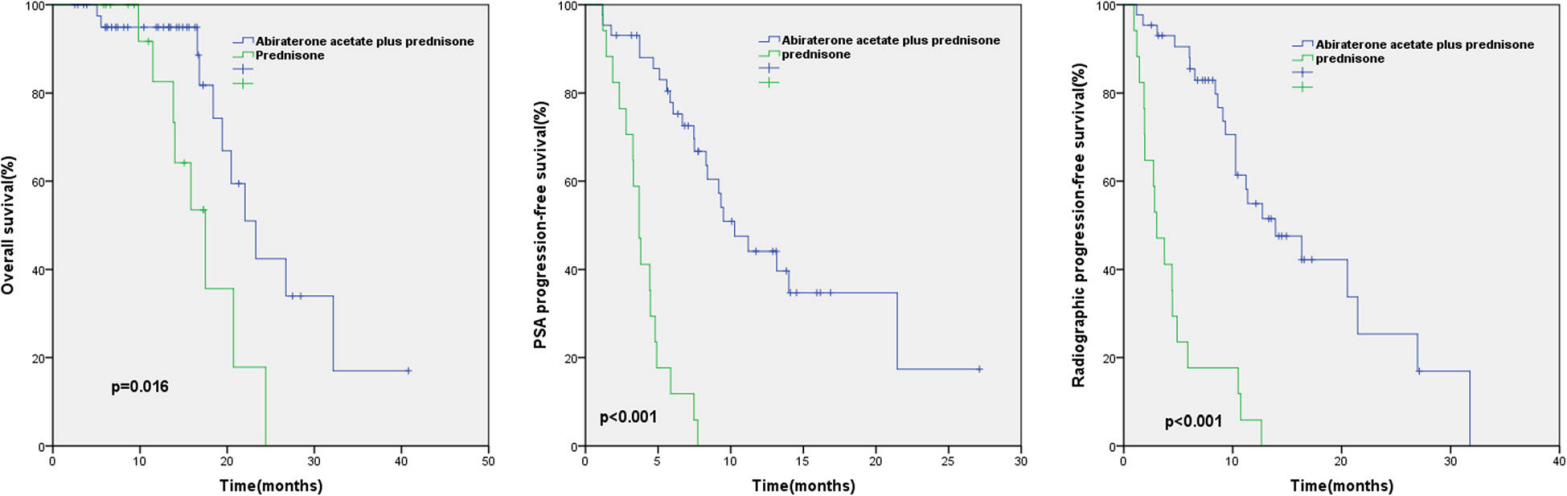
mCRPC patients in the AA + prednisone group had significantly longer survivals than those in prednisone group

The median PSA PFS was significantly longer for the AA + prednisone group than the prednisone-alone group (10.3 months [7.7–12.9]vs 3.0 months [1.7–4.4],*P* < 0.001), as was the median rPFS (13.9 months [8.4–19.5]vs 3.9 months [3.0–4.4],*P* < 0.001), and the median OS (23.3 months [18.8–27.7]vs 17.5 months [13.2–21.8],*P* = 0.016) (Fig. 1). The PSA response rate (≥50% decline) of the AA + prednisone group (27 of 43 patients [62.8%]) was more than five times that of the prednisone-alone group (2 of 17patients [11.8%]). Table 1 summarises the survival outcomes of patients in the AA + prednisone and prednisone-alone groups.

**Table 1:** Comparison of survival outcomes between the AA + prednisone and prednisone-alone groups

	Median OS, months(IQR)	Median PSA PFS,months(IQR)	Median rPFS, months(IQR)	PSA response rate
AA				
+ prednisone group	23.3 (18.8~ 27.7)	10.3 (7.7~ 12.9)	13.9 (8.4~ 19.5)	62.79% (27 of 43)
Prednisone-alone group	17.5 (13.2~ 21.8)	3.0 (1.7~ 4.4)	3.9 (3.0~ 4.4)	11.76% (2 of 17)
*P* value	0.016	< 0.001	< 0.001	< 0.001

Abbreviations: *AA* abiraterone acetate, *OS* overall survival, *PSA PFS PSA* progression free survival, *rPFS* radiographic progression free survival, *IQR* interquartile range

### Patient characteristics

In the AA-prednisone group (*n* = 43), the median age was 67 years (63–76). Of the 43 patients, 35 (81.4%) had ECOG-PS scores of 0 or 1,and 8 (18.6%) had an ECOG-PS of 2. Gleason scores were available for all 43 patients; 15 (34.88%) had Gleason scores of < 7 and 28 (65.2%) had 8–10, respectively. The baseline PSA at treatment initiation was 41.5 (range 15.9–239) ng/mL. All 43 patients had confirmed metastases; 42 (97.7%) had bone metastases, 17 (39.6%) had lymph node involvement, and 1 (2.3%) had lung metastases. Eleven patients (25.6%)were symptomatic prior to initiation of AA + prednisone therapy.

In the prednisone-alone group (*n* = 17), the median age was 67 years (62.5–74.5), 14 (82.4%) patients had ECOG-PS scores of 0 or 1, and 3 patients (17.6%) had ECOG-PS scores of 2. Seven patients (41.2%) had a Gleason score < 7 and 10 (58.8%) had scores between 8 and 10. The baseline PSA at treatment initiation was 46.6 (range 38–92.4)ng/dL. All 17 patients had confirmed bone metastases and 5 (29.4%)had lymph node involvement. As shown in Table 2, there were no significant differences in the clinical characteristics between patients in the AA + prednisone and prednisone-only groups in this study.

**Table 2:** Clinical characteristics of mCRPC patients (*n* = 60)

Parameters	AA group No. of patients(%)	Prednisone-alone group No. of patients(%)	*P* value
Age (median, IQR) years	67(63–76)	67(62.5~ 74.5)	0.8
ECOG PS			1.0
0	22(51.2%)	9(53.0%)	
1	13(30.2%)	5(29.4%)	
2	8(18.6%)	3(17.6%)	
PSA (median, IQR) μg/L	41.6 (15.9~ 239)	46.1(38.0~ 92.4)	0.7
Gleason Score			0.6
≤ 7	15(4.9%)	7(41.2%)	
> 7	28(65.1%)	10(58.8%)	
Metastatic site			
Bone metastasis	42(97.7%)	17(100%)	1
Lymph node metastasis	18(41.9%)	5(29.4%)	0.4
Lung metastasis	1(2.3%)	0(0%)	1
The number of bone metastasis			0.7
0	1(2.3%)	0(0%)	
1~ 3	13(30.2%)	4(23.5%)	
4~ 10	11(25.6%)	3(17.7%)	
> 10	18(41.9%)	10(58.8%)	
PSA PFS(percentage)	23(53.5%)	17(100%)	
rPFS(percentage)	23(53.5%)	8(47.1%)	
OS(percentage)	11(25.6%)	8(47.1%)	
Follow-up time (median, IQR) months	14.2 (7.2–18.4)	13.8 (9.0–17.4)	

Abbreviations: *HR* hazard ratio, *CI* confidence interval, *PSA* prostate-specific antigen, *ALP* alkaline phosphatase, *Hb* hemoglobin, *Alb* albumin, *ECOG PS* Eastern Collaborative Oncology Group performance status, *ADT* androgen deprivation therapy, *IQR* interquartile range

### Prognostic factors for chemotherapy-naive mCRPC patients treated with AA

**Fig. 2:**
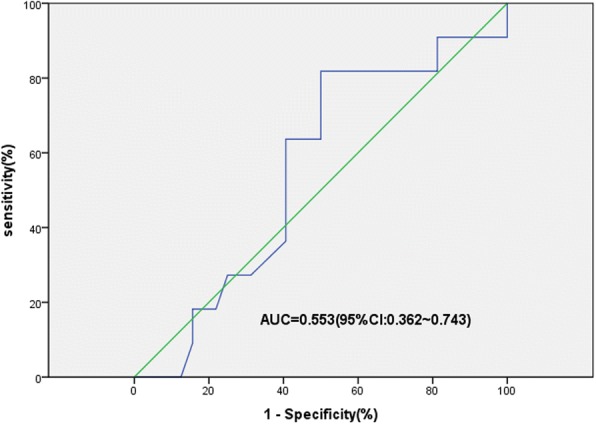
Receiver operating characteristic curve analysis of the optimal cut-off for the time from ADT to castration resistance

From the ROC curve analysis of the association between OS and the time from ADT to castration resistance, the optimal cut-off value was 18 months (Fig. 2). In univariate analysis, four variables were significant determinants of PSA PFS and OS and three were significant determinants of rPFS (Table 3). Several covariates were important predictors of outcomes in the multivariate survival models (Table 4). Short time from ADT to castration resistance (≤18 months) was a significant determinant of OS (hazard ratio [HR] = 12.8, 95% confidence interval [CI]: 2.0–83.1, *P* = 0.007); ECOG-PS score (HR = 2.6, 95% CI 1.2–5.5, *P* = 0.012), baseline PSA level (HR = 4.9, 95% CI: 1.0–23.0, *P* = 0.043), and short time from ADT to castration resistance (≤18 months) (HR = 3.4, 95% CI: 1.2–9.4, *P* = 0.02) were significant determinants of PSA PFS; and ECOG-PS (HR = 3.3, 95%CI: 1.5–7.2, *P* = 0.003) was also a significant determinant of rPFS.

**Table 3:** Univariate analyses of clinical parameters in mCRPC patients

Parameters	PSA PFS		OS		rPFS	
	HR (95% CI)	*P*-value	HR (95% CI)	P-value	HR (95% CI)	P-value
Age (years)	1.0 (0.9–1.0)	0.1	1.00 (0.9–1.1)	1.0	1.0 (0.9–1.0)	0.06
PSA (μg/L) (> 10 vs ≤10)	3.8 (0.9–16.2)	0.035	26.5 (0.01–49,948.6)	0.08	3.0 (0.7–12.7)	0.09
Gleason Score (> 7 vs ≤7)	0.8 (0.4–1.9)	0.65	1.07 (0.3–3.83)	0.92	0.9 (0.38–2.21)	0.84
ALP (≤120 vs > 120 U/L)	0.3 (0.1–0.7)	0.01	0.2 (0–0.9)	0.05	0.3 (0.09–0.66)	0.01
Hb(g/L)	1.0 (0.97–1.01)	0.34	1.0 (0.90–0.99)	0.029	1.0 (0.95–1.01)	0.14
Alb(g/L)	0.9 (0.8–1.0)	0.15	0.8 (0.6–1.0)	0.04	0.9 (0.8–1.0)	0.05
Time from ADT to castration resistance (≤18 vs > 18 months)	2.9 (1.2–7.1)	0.02	11.2 (2.2–57.7)	0.002	4.0 (1.5–10.7)	0.004
ECOG PS	2.9 (1.4–5.7)	0.004	1.6 (0.6–4.2)	0.34	3.6 (1.7–7.9)	0.001

Abbreviations: *HR* hazard ratio, *CI* confidence interval, *PSA* prostate-specific antigen, *ALP* alkaline phosphatase, *Hb* hemoglobin, *Alb* albumin, *ECOG PS* Eastern Collaborative Oncology Group performance status, *ADT* androgen deprivation therapy, *OS* overall survival, *PSA PFS PSA* progression free survival, *rPFS* radiographic progression free survival

**Table 4:** Multivariate analyses of clinical parameters in mCRPC patients

Parameters	PSA PFS		OS		rPFS	
	HR (95% CI)	P-value	HR (95% CI)	P-value	HR (95% CI)	P-value
PSA (μg/L) (> 10 vs ≤10)	4.9 (1.1–23.0)	0.04				
ALP (≤120 vs > 120 U/L)	0.6 (0.2–1.9)	0.37	0.1 (0–1.2)	0.07	0.4 (0.1–1.1)	0.08
Hb(g/L)			1.0 (0.9–1.0)	0.17		
Alb(g/L)			1.1 (0.8–1.5)	0.68		
Time from ADT to castration resistance (≤18 vs > 18 months)	3.4 (1.2–9.4)	0.02	12.8 (2.0–83.1)	0.007	2.6 (0.9~ 7.6)	0.08
ECOG PS	2.6 (1.2–5.5)	0.012			3.3 (1.5–7.2)	0.003

Abbreviations: *HR* hazard ratio, *CI* confidence interval, *PSA* prostate-specific antigen, *ALP* alkaline phosphatase, *Hb* hemoglobin, *Alb* albumin, *ECOG PS* Eastern Collaborative Oncology Group performance status, *ADT* androgen deprivation therapy, *OS* overall survival, *PSA PFS PSA* progression free survival, *rPFS* radiographic progression free survival

### Safety assessments

The most frequently reported grade 3 or 4 AE(reported in ≥3% of patients) in the AA + prednisone group were elevated alanine aminotransferase (ALT) levels (5 patients [11.6%]), hypokalaemia (4 patients [9.3%]), and hyperglycaemia (2 patients[4.7%]). In the prednisone group, the most frequently reported grade 3 or 4 AEs (reported in ≥3% of patients) were elevated ALT (2 patients [11.8%]), hypokalaemia, (2 patients [11.8%]), and hyperglycaemia(1 patient [5.9%]). None of the AEs resulted in discontinuation of therapy (Table 5).

**Table 5:** Grade 3 or 4 adverse events with an incidence of > 3%

Grade 3/4 AEs(reported in ≥3% of patients)	AA group	Prednisone-alone group
ALT increased	11.6% (5 of 43)	11.8% [2 of 17]
Hypokalaemia	9.3% (4 of 43)	11.8% [2 of 17]
Hyperglycaemia	4.7% (2 of 43)	5.9% [1 of 17]

Abbreviations: *ALT* Alanine aminotransferase, *AA* abiraterone acetate

## Discussion

The results of this study support the favourable safety and efficacy profile of AA for the treatment of Asian patients with chemotherapy-naive mCRPC. In addition, we observed that longer (≥18 months) duration of the ADT response was a significant determinant of survival of chemotherapy-naive mCRPC patients treated with AA. These results could serve to guide clinicians in determining whether patients with shorter (< 18 months) times from ADT to castration resistance might benefit more from chemotherapy than from AA.

An international collaborative study (COU-AA-302) confirmed that AA was effective for the treatment of chemotherapy-naive mCRPC patients, and significantly improved rPFS and OS with an acceptable AE profile [10]. In the current study, we demonstrated that AA is equally effective in the treatment of chemotherapy-naive mCRPC of Chinese ethnicity. To our knowledge, this is the first report of the long-term (follow-up > 12 months) efficacy and safety of AA in the treatment of Asian patients with chemotherapy-naive mCRPC.

The median OS and rPFS of our AA cohort (23.27 and 13.93 months, respectively) were shorter than those in the COU-AA-302 trial (34.7 and 16.5 months, respectively) [12] (Table 6). In this regard, a recent study reported a median OS of 18.1 months for chemotherapy-naive Asian mCRPC patients treated with AA, which is much shorter than the 34.7 months reported in the COU-AA-302 study [15]. Furthermore, a recent publication described the results of a bridging study of Asian patients in a randomised, double-blind, placebo-controlled phase 3 clinical trial of AA for mCRPC after docetaxel failure [18]. That study demonstrated a median PSA PFS of 5.5 months after treatment with AA + prednisone, which was also inferior to the PSA PFS outcome in the COU-AA-301 trial (median 8.5 months) [11]. We postulate that inclusion of chemotherapy-naive patients with poor prognostic features in our study could have accounted for the unsatisfactory survival results compared with the COU-AA-302 trial. Of note, in our study, chemotherapy-naive patients with an ECOG-PS score of 2, who were specifically excluded in the COU-AA-302 trial, had significantly inferior survival compared with patients with COG PS 0 and 1. The relative infrequency of post-AA treatment of our patient cohort compared with the COU-AA-302 trial cohort (11.6% versus 67% of patients, respectively) might also have contributed to the shorter survival time reported here (Table 7). Finally, the fact that our patient cohort included symptomatic patients, whereas the COU-AA-302 trial included only asymptomatic or mildly symptomatic patients, could also be a reason for the inferior survival in our study.

**Table 6:** Comparison of survival outcomesin the COU-AA-302 study and our study

	Median OS, months	Median PSA PFS, months	Median rPFS, months
Present study (AA plus prednisone group)	23.3	10.3	13.9
COU-AA-302 study	34.7	–	16.5

Abbreviations: *OS* overall survival, *PSA PFS PSA* progression free survival, *rPFS* radiographic progression free survival, *AA* abiraterone acetate

**Table 7:** Subsequent therapy for prostate cancer

	Percentage of patients (AA group in the present trial) (*N* = 43)	Percentage of patients (COU-AA-302 trial) (*N* = 546)
Patients with subsequent therapy	14.0%(6 of 43)	67% (365 of 546)
AA	2.3% (1 of 43)	13%(69 of 546)
Docetaxel	2.3% (1 of 43)	57%(311 of 546)
Estramustine phosphate	4.7% (2 of 43)	0
Radium-223	7% (3 of 43)	4%(20 of 546)

Abbreviations: *AA* abiraterone acetate

In the COU-AA-302 study, dose reduction and discontinuation because of treatment-related AEs were 7 and 7% of patients, respectively [13]. Patients In our study experienced very few AEs overall, and none resulted in discontinuation of drug therapy. Although the AEs may have been surveyed less vigorously in our study compared with clinical trials, AA appeared to be well tolerated by the Asian patients comprising our study.

In the present study, multivariate analysis indicated that shorter time (< 18 months) between ADT and castration resistance predicted worse survival outcome in the patients treated with AA + prednisone. Indeed, Cox regression analysis demonstrated that this was an independent prognostic factor for both OS and PSA PFS. Data from an earlier study showed that the duration of prior ADT is an independent prognostic factor for OS of chemotherapy-naive mCRPC patients(*P* = 0.002) [19]. However, the time from ADT to castration resistance is probably a better reflection of sensitivity to ADT. We should note that duration of prior hormonal therapy (HT) was defined as the total time to castration resistance plus the time with castration resistance on HT.

Studies of other HTs suggested that a short response to first-line ADT predicts a poor response to subsequent HT [20–22]. In a retrospective study of 436 patients with CRPC treated with secondary HT, the median duration of secondary HT treatment was longer for patients who received primary ADT for > 24 months than for those who received primary ADT for < 24 months (*P* < 0.0001) [20]. In addition, a report of 61 patients with chemotherapy-pretreated CRPC who were treated by AA revealed that duration of the ADT response was an independent predictor of OS(*P* = 0.006) [16]. This is consistent with a European consensus statement that short duration of ADT response could identify patients with increased risk of primary resistance to AR pathway-targeted therapies [23]. Polymorphisms in genes in the androgen metabolic pathway were reported to be significantly associated with time to progression on ADT, suggesting that shorter time to castration resistance may be associated with poorer response to ADT rather than to chemotherapy. This might also explain how the duration of response to previous ADT may influence the prognosis of CRPC patients treated with AA [24, 25].

Azad et al. [26] studied the prognostic significance of ECOG-PS and found that it was a significant predictor of PFS (*P* = 0.043), OS (*P* < 0.001), and PSA decline (*P* = 0.002). Our results showing that ECOG-PS was a significant determinant of rPFS and PSA PFS are consistent with that study.

It is interesting to note that treatment with AA early in the disease course may contribute to better survival. Considering the results of our multivariate analysis showing that ECOG-PS score was a significant determinant of both rPFS (HR = 2.606, *P* = 0.012) and PSA PFS (HR = 3.379, *P* = 0.02) and that baseline PSA level was a significant determinant of rPFS (HR = 4.914, *P* = 0.043), we postulate that better physical status may contribute to better survival outcomes of mCRPC patients treated with AA. We also found that just as comparative experimental results between COU-AA-301 trial and COU-AA-302 trial [10–13], the survival outcome of patients in our study was superior to that of Asian mCRPC patients treated with AA after docetaxel failure [18] in spite of being lack of OS because of short follow-up period (12.9 months) and limited number of observed death events (PSA PFS 10.27 months vs 5.5 months). These findings strengthen the rationale for the use of AA early in the clinical course of Asian mCRPC patients.

The study had some limitations. First, this study was a retrospective study which had a small sample size and was completed at a single centre. However, we consider this limitation would not influence the ability to capture the real survival outcome of AA in this study. Second, the number of patients in the AA + prednisone and prednisone-alone groups were unequal, which may have biased the results. Finally, the follow-up time was short and we do not know if the inferior survival outcomes reported here will continue at later follow-up times. In addition, another follow-up study will be planned in the future to evaluate the effect of sequential therapies.

The prognostic factors identified here should be validated, and we encourage others to continue the analysis of patients with mCRPC in different treatment settings, such as second-line chemotherapy, enzalutamide, or radium-223, for example. Such studies will aid in the development of optimised therapy sequences. Additionally, identifying patient subgroups who obtain the most benefit from AA could be important in maximising its cost-effectiveness [27–30].

## Conclusion

The AA plus prednisone treatment significantly prolonged PSA PFS, rPFS and OS in Asian patients with chemotherapy-naive mCRPC. The tolerance of patients was satisfactory and it is an effective and safe option for treating chemotherapy-naive mCRPC patients. Longer duration of ADT response was significantly associated with longer survival of chemotherapy-naive mCRPC patients treated with AA. This might help guide the selection of the best therapy for mCRPC.

## Additional file

Additional file 1: The raw data of mCRPC patients in this study. (XLSX 27 kb)

## Abbreviations

(PC): Prostate cancer
(PCWG2): Prostate Cancer Clinical Trials Working Group 2
AA: Abiraterone acetate
ADT: Androgen deprivation therapy
AEs: Adverse events
ALT: Alanine aminotransferase
ECOG-PS: Eastern Cooperative Oncology Group performance status
HR: Hazard ratio
IQR: Interquartile ranges
mCRPC: Metastatic castration-resistant prostate cancer
OS: Overall survival
PSA PFS: PSA progression-free survival
PSA: Prostate-specific antigen
ROC: Receiver operating characteristic
rPFS: Radiographic progression-free survival

## References

[1] Ferlay J, Shin HR, Bray F, Forman D, Mathers C, Parkin DM (2010). Estimates of worldwide burden of cancer in 2008: GLOBOCAN 2008. Int J Cancer.

[2] Sharifi N, Dahut WL, Steinberg SM (2005). A retrospective study of the time to clinical endpoints for advanced prostate cancer. BJU Int.

[3] Tannock IF, de Wit R, Berry WR (2004). Docetaxel plus prednisone or mitoxantrone plus prednisone for advanced prostate cancer. N Engl J Med.

[4] Petrylak DP, Tangen CM, Hussain MH (2004). Docetaxel and estramustine compared with mitoxantrone and prednisone for advanced refractory prostate cancer. N Engl J Med.

[5] de Bono JS, Oudard S, Ozguroglu M (2010). Prednisone plus cabazitaxel or mitoxantrone for metastatic castration-resistant prostate cancer progressing after docetaxel treatment: a randomised open-label trial. Lancet.

[6] Basch E, Loblaw DA, Oliver TK (2014). Systemic therapy in men with metastatic castration-resistant prostate cancer:American Society of Clinical Oncology and Cancer Care Ontario clinical practice guideline. J Clin Oncol.

[7] Attard G, Reid AH, A’Hern R (2009). Selective inhibition of CYP17 with abiraterone acetate is highly active in the treatment of castration-resistant prostate cancer. J Clin Oncol.

[8] Ang JE, Olmos D, de Bono JS (2009). CYP17 blockade by abiraterone: further evidence for frequent continued hormone-dependence in castration-resistant prostate cancer. Br J Cancer.

[9] Attard G, Reid AH, Yap TA (2008). Phase I clinical trial of a selective inhibitor of CYP17, abiraterone acetate, confirms that castration-resistant prostate cancer commonly remains hormone driven. J Clin Oncol.

[10] de Bono JS, Logothetis CJ, Molina A (2011). Abiraterone and increased survival in metastatic prostate cancer. N Engl J Med.

[11] Fizazi K, Scher HI, Molina A (2012). Abiraterone acetate for treatment of metastatic castration-resistant prostate cancer: final overall survival analysis of the COU-AA-301 randomised, double-blind, placebo-controlled phase 3 study. Lancet Oncol.

[12] Ryan CJ, Smith MR, de Bono JS (2013). Abiraterone in metastatic prostate cancer without previous chemotherapy. N Engl J Med.

[13] Ryan CJ, Smith MR, Fizazi K (2015). Abiraterone acetate plus prednisone versus placebo plus prednisone in chemotherapy-naive men with metastatic castration-resistant prostate cancer (COU-AA-302): final overall survival analysis of a randomised, double-blind, placebo-controlled phase 3 study. Lancet Oncol.

[14] Clayton R, Wu J, Heng DY (2014). A multicentre analysis of abiraterone acetate in Canadian patients with metastatic castration-resistant prostate cancer. Can Urol Assoc J = Journal de l'Association des urologues du Canada.

[15] Poon DM, Chan K, Lee SH (2016). Abiraterone acetate in metastatic castration-resistant prostate cancer - the unanticipated real-world clinical experience. BMC Urol.

[16] Davies RS, Smith C, Button MR (2015). What predicts minimal response to Abiraterone in metastatic castrate-resistant prostate Cancer?. Anticancer Res.

[17] Scher HI, Halabi S, Tannock I (2008). Design and end points of clinical trials for patients with progressive prostate cancer and castrate levels of testosterone: recommendations of the prostate Cancer clinical trials working group. J Clin Oncol.

[18] Sun Y, Zou Q, Sun Z (2016). Abiraterone acetate for metastatic castration-resistant prostate cancer after docetaxel failure: a randomized, double-blind, placebo-controlled phase 3 bridging study. Int J Urol.

[19] Bellmunt J, Kheoh T, Yu MK, et al. Prior endocrine therapy impact on Abiraterone acetate clinical efficacy in metastatic castration-resistant prostate Cancer: post-hoc analysis of randomised phase 3 studies. Eur Urol. 2015. 10.1016/j.eururo.2015.10.021 PMC5465643 26508309

[20] Nakabayashi M, Werner L, Oh WK, Regan MM, Kantoff PW, Taplin ME (2011). Secondary hormonal therapy in men with castration-resistant prostate cancer. Clin Genitourin Cancer.

[21] Nakabayashi M, Xie W, Regan MM, Jackman DM, Kantoff PW, Oh WK (2006). Response to low-dose ketoconazole and subsequent dose escalation to high-dose ketoconazole in patients with androgen-independent prostate cancer. Cancer.

[22] Loriot Y, Eymard JC, Patrikidou A (2015). Prior long response to androgen deprivation predicts response to next-generation androgen receptor axis targeted drugs in castration resistant prostate cancer. Eur J Cancer.

[23] Fitzpatrick JM, Bellmunt J, Fizazi K (2014). Optimal management of metastatic castration-resistant prostate cancer: highlights from a European expert consensus panel. Eur J Cancer.

[24] Ross RW, Oh WK, Xie W (2008). Inherited variation in the androgen pathway is associated with the efficacy of androgen-deprivation therapy in men with prostate cancer. J Clin Oncol.

[25] Yang M, Xie W, Mostaghel E (2011). SLCO2B1 and SLCO1B3 may determine time to progression for patients receiving androgen deprivation therapy for prostate cancer. J Clin Oncol.

[26] Azad AA, Eigl BJ, Leibowitz-Amit R (2015). Outcomes with abiraterone acetate in metastatic castration-resistant prostate cancer patients who have poor performance status. Eur Urol.

[27] Liancheng Fan, Rui Wang, Chenfei Chi, Wen Cai, Yong Zhang, Hongyang Qian, Xiaoguang Shao, Yanqing Wang, Fan Xu, Jiahua Pan, Yinjie Zhu, Xun Shangguan, Lixin Zhou, Baijun Dong, Wei Xue. Systemic immune-inflammation index predicts the combined clinical outcome after sequential therapy with abiraterone and docetaxel for metastatic castration-resistant prostate cancer patients. The Prostate. 2018;78(4):250-56. 10.1002/pros.23465 29285775

[28] Baijun Dong, Liancheng Fan, Yanqing Wang, Chenfei Chi, Xiaowei Ma, Rui Wang, Wen Cai, Xiaoguang Shao, Jiahua Pan, Yinjie Zhu, Xun Shangguan, Zhixiang Xin, Jianian Hu, Shaowei Xie, Xiaonan Kang, Lixin Zhou, Wei Xue. Influence of abiraterone acetate on neuroendocrine differentiation in chemotherapy-naive metastatic castration-resistant prostate cancer. The Prostate. 2017;77(13):1373-380. 10.1002/pros.23397 28804908

[29] Liancheng Fan, Yanqing Wang, Chenfei Chi, Jiahua Pan, Shangguan Xun, Zhixiang Xin, Jianian Hu, Lixin Zhou, Baijun Dong, Wei Xue. Chromogranin A and neurone-specific enolase variations during the first 3 months of abiraterone therapy predict outcomes in patients with metastatic castration-resistant prostate cancer. BJU International. 2017;120(2):226-32. 10.1111/bju.13781 28107595

[30] Liancheng Fan, Xiao Wang, Chenfei Chi, Yanqing Wang, Wen Cai, Xiaoguang Shao, Fan Xu, Jiahua Pan, Yinjie Zhu, Xun Shangguan, Zhixiang Xin, Jianian Hu, Shaowei Xie, Rui Wang, Lixin Zhou, Baijun Dong, Wei Xue. Prognostic nutritional index predicts initial response to treatment and prognosis in metastatic castration-resistant prostate cancer patients treated with abiraterone. The Prostate. 2017;77(12):1233-241. 10.1002/pros.23381 28752926

